# Establishing an expert consensus for the operational definitions of asthma-associated infectious and inflammatory multimorbidities for computational algorithms through a modified Delphi technique

**DOI:** 10.1186/s12911-021-01663-y

**Published:** 2021-11-08

**Authors:** Jungwon Yoon, Heather Billings, Chung-Il Wi, Elissa Hall, Sunghwan Sohn, Jung Hyun Kwon, Euijung Ryu, Pragya Shrestha, Hongfang Liu, Young J. Juhn

**Affiliations:** 1 Department of Pediatric and Adolescent Medicine, Mayo Clinic, Rochester, MN USA; 2 Precision Population Science Lab, Mayo Clinic, Rochester, MN USA; 3 Department of Pediatrics, Myongji Hospital, Goyang-si, South Korea; 4 Office of Applied Scholarship and Education Science, Mayo Clinic, Rochester, MN USA; 5 Division of Digital Health Sciences, Mayo Clinic, Rochester, MN USA; 6 Department of Pediatrics, Korea University College of Medicine, Seoul, South Korea; 7 Division of Biomedical Statistics and Informatics, Mayo Clinic, Rochester, MN USA; 8 Department of Pediatric and Adolescent Medicine and Internal Medicine, Mayo Clinic, 200 1st Street SW, Rochester, MN 55905 USA

**Keywords:** Delphi, Asthma, Multimorbidities, Electronic health records, Natural language processing

## Abstract

**Background:**

A subgroup of patients with asthma has been reported to have an increased risk for asthma-associated infectious and inflammatory multimorbidities (AIMs). To systematically investigate the association of asthma with AIMs using a large patient cohort, it is desired to leverage a broad range of electronic health record (EHR) data sources to automatically identify AIMs accurately and efficiently.

**Methods:**

We established an expert consensus for an operational definition for each AIM from EHR through a modified Delphi technique. A series of questions about the operational definition of 19 AIMS (11 infectious diseases and 8 inflammatory diseases) was generated by a core team of experts who considered feasibility, balance between sensitivity and specificity, and generalizability. Eight internal and 5 external expert panelists were invited to individually complete a series of online questionnaires and provide judgement and feedback throughout three sequential internal rounds and two external rounds. Panelists’ responses were collected, descriptive statistics tabulated, and results reported back to the entire group. Following each round the core team of experts made iterative edits to the operational definitions until a moderate (≥ 60%) or strong (≥ 80%) level of consensus among the panel was achieved.

**Results:**

Response rates for each Delphi round were 100% in all 5 rounds with the achievement of the following consensus levels: (1) Internal panel consensus: 100% for 8 definitions, 88% for 10 definitions, and 75% for 1 definition, (2) External panel consensus: 100% for 12 definitions and 80% for 7 definitions.

**Conclusions:**

The final operational definitions of AIMs established through a modified Delphi technique can serve as a foundation for developing computational algorithms to automatically identify AIMs from EHRs to enable large scale research studies on patient’s multimorbidities associated with asthma.

**Supplementary Information:**

The online version contains supplementary material available at 10.1186/s12911-021-01663-y.

## Background

Asthma is the most common chronic illness of childhood, representing one of the five most burdensome chronic diseases in US adults [1–3]. Our group and others demonstrated asthma’s impact on the risks of a broad range of infectious and inflammatory diseases, *namely asthma-associated infectious and inflammatory multimorbidities (AIMs)* as an under-recognized health threat to adults and children with asthma [4–26]. The Centers for Disease Control and Prevention terms asthma as an independent risk factor for invasive pneumococcal diseases. At present, the 2008 US and 2014 Canadian pneumococcal vaccine policies recommend a single dose of 23-valent pneumococcal polysaccharide vaccine (PPV-23) to adults with asthma ages 19–64 years [27–29]. A recent prospective cohort study showed that asthma’s impact on the risk of any infection as measured by a population attributable risk percent was similar to that of diabetes mellitus(DM) (2.2% vs. 2.9%, respectively) [8]. Impairment of both innate and adaptive immunity is the currently proposed mechanism for this association [9]. To mitigate the risks and outcomes of AIMs through clinical care and research, it is necessary to develop innovative strategies enabling identification and characterization of a subgroup of asthmatic children at risk of AIMs at a population level, especially studies requiring precision such as mechanism studies.

For example, to study the nature of the association of asthma with the risk of pneumonia, one must identify pneumonia by more than the inherently limited International Statistical Classification of Diseases (ICD) codes. The additional use of practical, predetermined criteria derived from guidelines and expert panels leveraging a broad range of EHR data sources is crucial for accurate and efficient identification in EHR-based studies. This includes the free text in a physician diagnosis, key terms referring to pneumonia in chest x-rays, and clinical notes. This type of manual chart review can be done for smaller studies, but becomes burdensome when applied to large, population-based studies with the goal of investigating numerous AIMs. Our group has developed and applied multiple computational algorithms (eg, natural language processing [NLP] algorithms) to automate the chart review process in regards to the two existing asthma criteria, asthma prognosis, and other asthma outcomes [30–33]. Those algorithms fully leverage EHRs in a way that effectively and efficiently phenotypes asthma, making asthma care and research scalable [30–32]. On the other hand, despite the significance of AIMs as a major health threat to people with asthma, computational algorithms for identifying AIMs are largely unavailable due to the lack of operational definitions which are critical for developing computational algorithms to automate the chart review process.

Deriving operational definitions based on the consensus of expert panels facilitates transparency and interpretability of downstream computational algorithms. Computational algorithms for a broad range of health conditions developed by different institutions using different approaches are available through eMERGE [34] and PheKB [35, 36]. However, very few studies report the development process of a rigorous operational definition for computational algorithms that can be reused systematically in a new area [37].

Herein we demonstrate the process and the results of establishing an operational definition for each AIM for the pediatric population through consensus building among internal and external experts using a modified Delphi method. The Delphi method relies on a group of experts to provide sequential levels of anonymous responses and feedback through a series of questionnaires to reduce range of responses and arrive at a predetermined level of consensus. This consensus building method is becoming a popular means through which to generate operational definitions and practice guidelines, as evident through recent work published in the fields of Otolaryngology, Pediatric Critical Care, and Geriatrics to name but a few [38–42].

## Methods

The Delphi method has a myriad of applications and is often used in health services research, providing controlled feedback and systematic progression toward consensus among a panel of experts during completion of a series of voting rounds [43–46]. A modified process eliminates the initial open-ended questionnaire phase in lieu of a pre-population of statements that are reviewed and voted on by a panel of experts [47–49]. This modified Delphi method for consensus building serves as the methodological framework for researchers to develop and apply computational algorithms for AIMs.

### Participants

A core team of five clinicians and scientists drafted the initial set of operational definitions. The internal expert panel consisted of eight physicians from a single institution practicing in the fields of Rheumatology (TM, AO), Infectious Disease (CH, ER), Gastroenterology (IA, MG) and Allergy (MP, AR). The five-member external panel of experts consisted of physicians practicing in the fields of Family Medicine/Epidemiology, Infectious Disease, Rheumatology, Pediatric Critical Care, and Gastroenterology all within the United States (see the Acknowledgement section). Criteria for panel inclusion included national recognition as an expert as evidenced through multiple peer reviewed publications in their field, presentations at national conferences, and leadership roles in professional societies. Participants were informed that participation in the project would require them to complete multiple Delphi rounds during which they would complete an online questionnaire, which would take 5–7 min. See Table 1 for detailed demographics of panel members.

**Table 1:** Participants’ demographic information

Characteristics	Number
*Sex*	
Male	8
Female	5
*Academic ranking*	
Professor	5
Associate Professor	2
Assistant Professor	5
Instructor	1
*Medical doctor specialty*	
Rheumatology	3
Infectious Disease	3
Gastroenterology	3
Allergy	2
Family medicine/epidemiology	1
Pediatric critical care	1
*Number of years post-residency training*	
0–4	1
5–10	6
> 10	6

### Questionnaire development

**Fig. 1:**
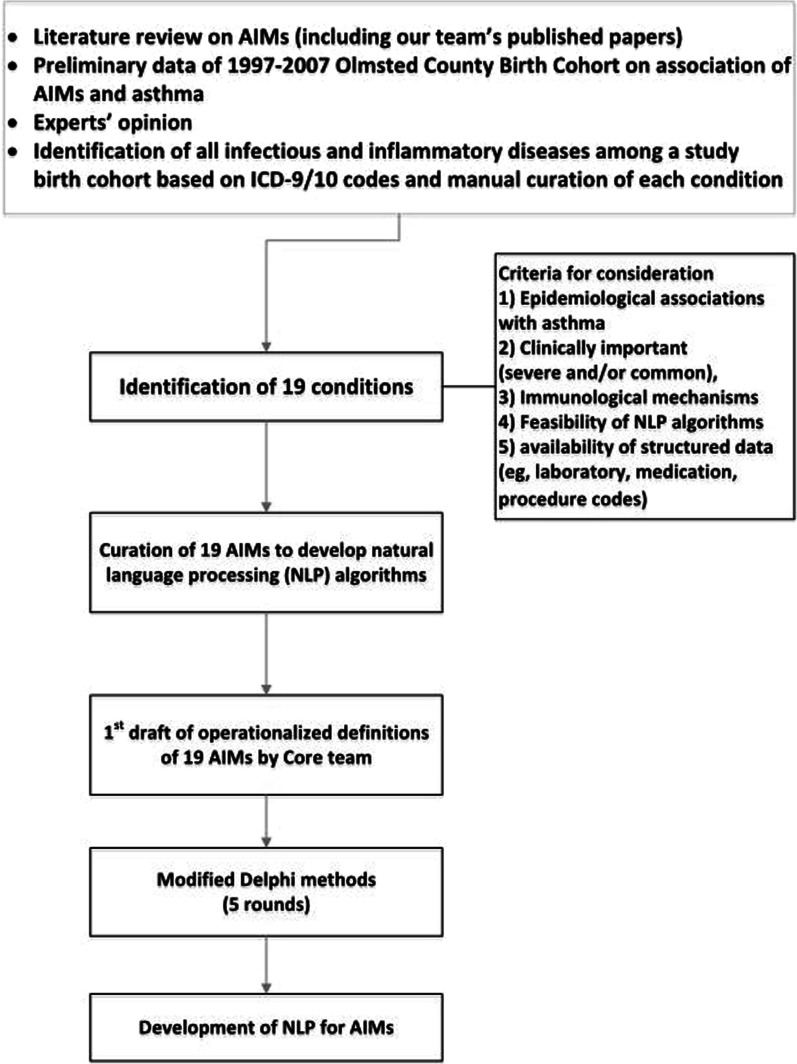
Overall process for identifying 19 AIMs and their operational definitions

In order to posit the list of 19 potential AIMs and their operational definitions, the core team conducted (1) literature reviews per AIM and (2) a manual review of all ICD-9/10 codes for 24,003 electronic health records of children who were born in Olmsted County, Minnesota, between 1997 and 2016. These definitions were categorized within infectious disease conditions (11) and inflammatory disease conditions (8) (Fig. 1).

**Fig. 2:**
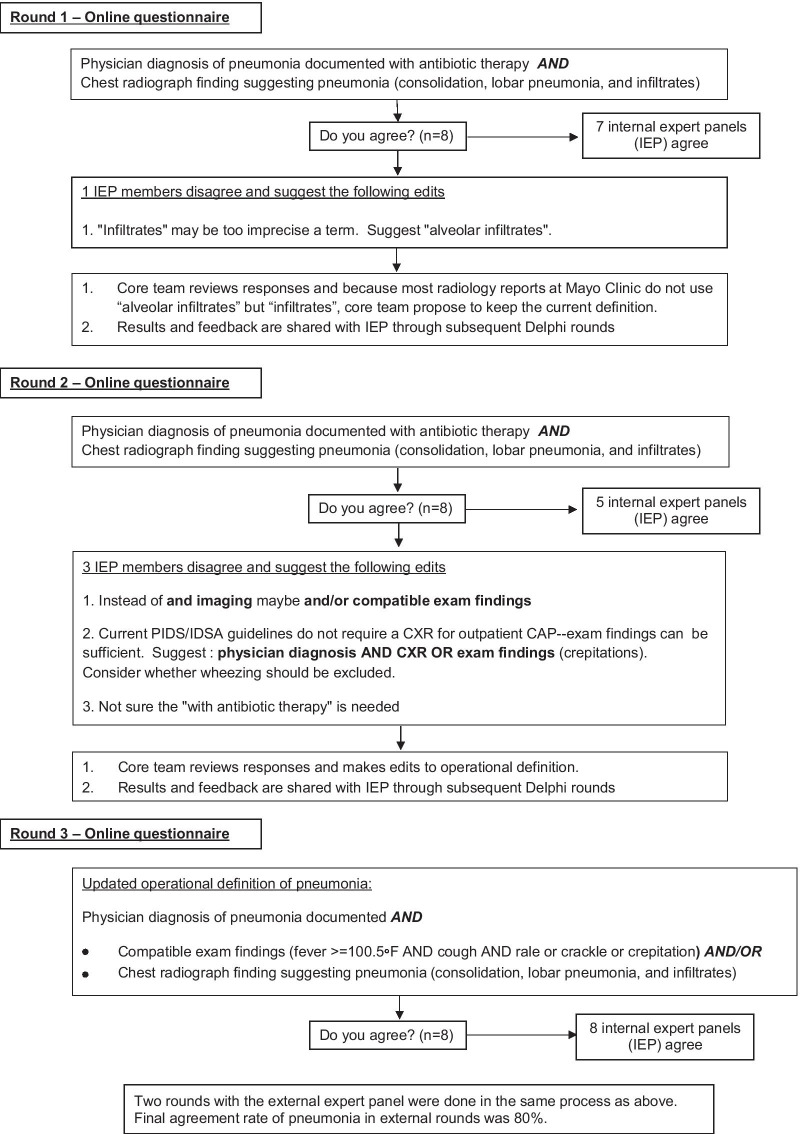
Example of Delphi process and iterative submission of operational definitions for AIMs (e.g., Pneumonia). Three internal and two external rounds were completed sequentially

The definitions were composed via an online questionnaire distributed through email and administered using Qualtrics software (version 2017, Provo, UT). Each operational definition included one or more proposed criteria statements linked by a conjunction such as “and”, or “or”. Respondents were asked to respond Yes or No to the prompt “Do you agree with this?” for each of the operational definitions (Fig. 2). Respondents were instructed to keep the following questions in mind:

1. Is the operational definition of sufficient clinical value to warrant inclusion?
2. Is the wording of the operational definition clear and precise to avoid misinterpretation?

If the response was No, respondents were prompted to suggest specific changes in the subsequent free text space. All responses and comments were reviewed, and edits were incorporated into the next iteration of definitions. Figure 2 shows an example of the survey process. Moderate consensus was defined as 60–79% agreement among respondents, and strong consensus was defined as ≥ 80% agreement among respondents for each AIM.

### Internal expert panel (IEP) voting rounds

The IEP members were given 7 days to complete the online questionnaire. Three follow-up emails were sent to non-respondents on the 3rd, 4th and 7th day which resulted in completion of the survey by all 8 IEP members. Core team members reviewed all response data and drafted modified operational definitions as necessary. These modified definitions were then resubmitted for review through an online questionnaire during the next Delphi round until consensus (≥ 60%) was achieved. Rounds two and three followed an identical process including the 3 subsequent reminders.

### External expert panel (EEP) voting rounds

A five-member External Expert Panel (EEP) was enlisted to confirm consensus of the 19 operational definitions, which had achieved moderate or greater consensus among the IEP members. Two Delphi voting rounds were conducted following the same process as with the IEP until ≥ 60% agreement. Additional file 1: Fig. 1 shows the questionnaire used at the 2nd round for EEP.

This project was approved by the Mayo Clinic Institutional Review Board (#14-009934).

## Results

### Definitions of AIMs and response rate

In this study, 19 operational definitions were generated by a core team and then updated based on three sequential rounds with 8 IEP members, and two rounds with 5 EEP members from various institutions (Table 2). Response rate from participants was 100% for each round.

**Table 2:** Final operational definition of 19 asthma-associated infectious and inflammatory comorbidities

Infectious diseases	EHR sources
1. *Invasive Bacterial Infection: A AND B* A: Any pathogenic bacteria isolated from normally sterile body fluid including blood, CSF, pleural fluid, pericardia fluid, peritoneal fluid, or synovial fluid, B. Any physician diagnosis of sepsis, bacteremia, meningitis, encephalitis, mastoiditis, brain abscess, pneumonia, cellulitis, osteomyelitis, septic arthritis, pleuritis, or pericarditis, and pyelonephritis documented in medical records related to bacteria source cultured	Lab result, Clinical note (Diagnosis)
2. *Frequent Streptococcus Pyogenes Upper Respiratory Infection: A AND B AND C* A. Physician diagnosis of sore throat, pharyngitis and tonsillitis B. Throat swab test (rapid antigen detection test (RADT), Streptococcus pyogenes culture, or PCR [Polymerase chain reaction]) C. 3 or more episodes within 12 months	Lab result, Clinical note (Diagnosis)
3. *Pneumonia: A AND (B AND/OR C)* A. Physician diagnosis of pneumonia B. Compatible exam findings (fever > = 100.5◦F AND cough AND rale or crackle or crepitation) C. Chest radiograph finding suggesting pneumonia (consolidation, lobar pneumonia, and infiltrates)	Clinical note (Diagnosis, History of present illness, Physical examination), Chest X-ray finding
4. *Recurrent or Persistent Otitis Media* CPT (Current Procedural Terminology) codes for tympanostomy tube placement (surrogate marker for either persistent or recurrent otitis media during childhood)	CPT codes
5. *Recurrent or Persistent Infectious Sinusitis: A AND (B AND/OR C)* A. 4 or more episodes of Physician diagnosis of sinusitis documented with antibiotic prescription over 12 months B. Sinus CT findings suggestive of sinus opacification or air/fluid level C. Sinus surgery	Clinical note (Diagnosis), Computerized Tomography (CT) finding, Operational note
6. *Bordetella Pertussis* Polymerase Chain Reaction (PCR) + for Bordetella pertussis from the upper respiratory tract	Lab result
7. *Breakthrough Varicella Infection: A AND/OR B* A. Physician diagnosis of Varicella (chickenpox) B. Positive lab result (PCR +) of varicella infection occurred 42 days after varicella vaccination (excluding non-vaccinated children)	Lab result, Clinical note (Diagnosis)
8. *Zoster (Shingles): A AND (B AND/OR C)* A. Physician diagnosis of zoster B. Positive lab result (PCR +) C. Anti-viral medication for Varicella zoster virus (e.g. acyclovir)	Lab result, Clinical note, Medication prescription
9. *Urinary Tract Infection (UTI): Urinary test results supporting the evidence of UTI as follows; A AND B* A. Recovery of any organisms from a suprapubic specimen, at least 50 000 colony-forming units per milliliter (CFUs/mL) from a catheterized specimen, or at least 100 000 CFUs/mL from a clean-catch specimen B. At least 10 white blood cells per microliter from an unspun specimen examined using a counting chamber or at least 5 white blood cells per high power field from a centrifuged specimen	Lab result
10. *Skin Fungal Infection: A AND/OR B* A. Physician diagnosis of any skin fungal infection with antifungal therapy B. Fungal culture or fungal smear positive	Lab result, Clinical note (Diagnosis)
11. *Clinically Significant Viral Infection confirmed by lab: A AND B* *A*. A physician diagnosis of respiratory or gastrointestinal viral infection B. PCR + or culture + test for respiratory or gastrointestinal virus infection	Lab result, Clinical note (Diagnosis)
*Inflammatory diseases*	
12. *Celiac Disease (CD): A AND ([B AND C] AND/OR [D AND E])* A. Physician diagnosis of Celiac disease documented at least once by gastroenterologist B. Positive CD serology markers (TTG lgA > 10 higher than normal) C. EMA positivity or DGA positively D. TTG IgA positivity E. Histologic findings (increased in IEL, villous atrophy, crypts hyperplasia)	Lab result, Clinical note (Diagnosis), Endoscopy finding
13. *Kawasaki Disease* Physician diagnosis of Kawasaki disease documented at least once by infectious disease, cardiology, or rheumatology specialist	Clinical note (Diagnosis)
14. *Appendicitis: A OR (B AND C)* A. Surgeon's diagnosis in operation note (excluding incidental appendectomy or normal appendix) B. Physician diagnosis of appendicitis C. Imaging study suggestive of appendicitis	Clinical note (Diagnosis), Operational note, CT or Ultrasound finding
15. *Autoimmune Thyroiditis* Physician diagnosis of autoimmune thyroiditis documented at least twice in a 6 month or greater span including endocrinologist's diagnosis at least once	Clinical note (Diagnosis)
16. *Diabetes Type 1* Physician diagnosis of Type 1 Diabetes documented at least twice in a 6 month or greater span including endocrinologist's diagnosis at least once	Clinical note (Diagnosis)
17. *Diabetes Type 2* Physician diagnosis of Type 2 Diabetes documented at least twice in a 6 month or greater span including endocrinologist's diagnosis at least once	Clinical note (Diagnosis)
18. *Inflammatory Bowel Disease (IBD; Crohn's Disease (CD), Ulcerative Colitis (UC))* Physician diagnosis of IBD, CD, or UC documented at least twice in a 6 month or greater span including gastroenterologist's diagnosis at least once	Clinical note (Diagnosis)
19. *Juvenile Rheumatoid Arthritis (JRA), Juvenile idiopathic Arthritis (JIA), or Rheumatoid Arthritis (RA)* Physician diagnosis of JRA, JIA, and RA documented at least twice in a 6 month or greater span including a rheumatologist's diagnosis at least once	Clinical note (Diagnosis)

### Percentage agreement rate for each AIM

Percentage agreement rate for each definition of AIMs is summarized in Table 3.

**Table 3:** The consensus level attained for each of the 19 operational definitions of asthma associated infectious and inflammatory multimorbidities (AIMs)

	Total mean (SD)% of agreement (n = 13)	Final % of IEP that agreed with the definition (n = 8)	Final % of EEP that agreed with the definition (n = 5)
Invasive bacterial infection	91 (9)	88	100
Frequent Streptococcus pyogenes upper respiratory infection	87 (8)	88	80
Pneumonia	82 (14)	100	80
Recurrent/persistent otitis media	87 (19)	100	100
Recurrent/persistent infectious sinusitis	80 (17)	88	100
Bordetella pertussis	98 (6)	100	100
Breakthrough varicella infection	94 (9)	88	100
Zoster (Shingles)	86 (17)	100	80
Urinary Tract Infection	89 (7)	88	100
Skin Fungal Infection	85 (10)	100	80
Viral Infection confirmed by lab	85 (21)	100	100
Celiac disease	83 (24)	100	100
Kawasaki disease	84 (11)	75	80
Appendicitis	98 (6)	88	100
Autoimmune thyroiditis	82 (25)	88	80
Diabetes type 1	90 (17)	88	100
Diabetes type 2	90 (17)	88	100
Inflammatory bowel disease	77 (18)	100	80
JRA, JA, RA	84 (16)	100	100

JRA, Juvenile Rheumatoid Arthritis; JA, Juvenile idiopathic Arthritis; RA, Rheumatoid Arthritis

Consensus levels achieved are as follows: (1) Internal panel consensus: 100% for 8 definitions, 88% for 10 definitions, and 75% for 1 definition, (2) External panel consensus: 100% for 12 definitions and 80% for 7 definitions. In total, 18 of 19 definitions achieved strong consensus (≥ 80%) in both internal and external rounds. The single definition that did not reach strong consensus was regarding Kawasaki disease (KD) which achieved a consensus rating of 75% by the final internal round. Insight into this lower consensus comes from the comments of the 2 panelists who didn’t agree with the definition. The comments are as follows: “How will you handle a “possible” or “rule out” diagnosis? I think the IVIG (Intravenous immunoglobulin) treatment requirement is helpful” and “I agree with the definition, but I am not sure if this happens in practice (e.g. I don’t believe specialists are always consulted in the inpatient setting)”. Treatment condition (e.g. IVIG) was initially included in the definition but, the panel proposed to take it out and the core team of experts accepted and updated the definition after the second internal round. Thus, 7 out of 8 panels actually agreed with the definition, but one panelist selected “No” solely due to the electronic format (the panelist had a comment to offer and could only have access to a texting box if they selected “No”.

### Feedback from the panels and process of modifications

The reasons for the disagreement from expert panelists were summarized as follows: (1) requiring more confirmative methods (53%)—e.g. specialist diagnosis and laboratory findings, (2) definition is too narrow (22%), (3) wording of the operational definition needs clarification (19%), (4) clarifying the condition of “period” by stating a definitive span of time, in the cases of recurrent or chronic disease (4%), and (5) others—e.g. editorial error. The core team reviewed the suggestions made by the internal and/or external panelists with consideration given to the following points: (1) existing literature on the operational definition of each AIM, (2) balance between sensitivity and specificity of each condition, (3) generalizability when used at other institutions, and (4) feasibility of development of computational algorithm.

## Discussion

Response rates for each Delphi round were 100% in all 5 rounds. Using a modified Delphi technique, we achieved strong consensus (≥ 80%) for operational definitions of all AIMs except one (75% at the final internal round), which can be applicable to computational algorithms. Overall, this process adds accuracy and reliability to studies concerning the association between asthma and AIMs.

At the end of three sequential rounds, 7 to 8 of the eight internal panelists (depending on the specific AIM being evaluated) reached agreement on the final operational definition of each AIM resulting in an 88–100% consensus. For example, at the end of the first round, 4 AIMs had achieved a consensus of less than 70%, but through the reiteration process (e.g. feedback and modification), we reached a moderate to strong consensus (≥ 75%) for all AIMs. Upon sending the updated definitions to external panelists, 5 AIMs still had a consensus of less than 70% at the end of the second and final round, including recurrent otitis media, recurrent infections sinusitis, autoimmune thyroiditis, diabetes type 1 and 2. Of these 5, we modified the definitions of three inflammatory diseases to make them more specific, but proposed to keep the definition of the two infectious diseases by making further clarifications. As a result, 4 to 5 out of 5 panelists (80–100%) reached agreement on the final definitions of each AIM. Given their different specialties and practice settings, the feedback from the group of external experts was very helpful for ascertaining the generalizability of these operationalized definitions in other study settings.

This novel study is the first to use a modified Delphi method to construct operational definitions for each AIM enabling us to develop computational algorithms to identify AIMs from EHRs. After considering literature, feasibility (data accessibility, specificity (for mechanism study), and generalizability (for implementing to other institutions), the balance between specificity and sensitivity of each definition was decided by the core team in response to panel feedback with updating of definitions as deemed reasonable.

The main concern of the panelists was the need for other diagnostic methods in the operational definition. For example, the operational definitions of six inflammatory diseases including Kawasaki disease, autoimmune thyroiditis, diabetes type 1 and 2, inflammatory bowel disease, and the arthritis group (Juvenile Rheumatoid Arthritis, Juvenile idiopathic Arthritis, or Rheumatoid Arthritis) were defined only by physician diagnosis including a specialist’s diagnosis at least once. In the initial operational definition, more than two diagnoses by a physician were included, but in accordance with feedback from the panelists, this definition was modified. It now reads as requiring the diagnosis of specialist at least once and this is deemed reasonable for confirmation as these diseases are generally confirmed by specialists who see the patient after referral from a primary care physician or clinician at another medical institution. Furthermore, the precision of some of the AIMs was improved via the panelist’s suggestions of adding a laboratory test or treatment condition (e.g. urinary tract infection (UTI)). For example, historically, a varicella diagnosis was made clinically. Due to the varicella vaccine, varicella symptoms become milder with fewer lesions. In turn, this has caused clinicians to become more conservative in making a diagnosis of varicella. Currently, evaluation and management of varicella cases in outpatient settings is still primarily based on clinical ground. Unless there is a public health concern viral testing is infrequently ordered. Thus, we proposed the operational definition as, physician diagnosis of varicella AND/OR positive laboratory test. Having said that, our group has a project that focuses on understanding the immune mechanisms of AIMs as it relates to asthma, and therefore we took a generally conservative stance of keeping the definition even as it increases specificity at the cost of potentially reducing sensitivity. Depending on the scope of the study, other investigators can revise these definitions for each AIM, tailoring it to their particular study (e.g. balancing sensitivity vs. specificity).

For some AIMs, the core team initially proposed using only structured data, which was agreed upon by panelists, such as tympanostomy placement as a surrogate marker for recurrent or persistent otitis media. Since a child can have persistent or recurrent otitis media without having tympanostomy, the proposed definition can only identify those children who had tympanostomy tubes placed, which may increase specificity, but lower sensitivity. However, since clinicians utilize various diagnostic terms when referring to recurrent or persistent ear infections, it is challenging to identify children with true recurrent or persistent ear infections, especially using a computer program like natural language processing (NLP) algorithm. A previous study addressed this problem by using Current Procedural Terminology (CPT) codes for tympanostomy tube placement to identify children with recurrent or persistent ear infections [50]. As ear infections are a common childhood malady and diagnosis/identification of ear infections in medical records are variable, we chose to be conservative in identifying recurrent or persistent ear infections. At any rate, it is worth noting that differential data sources and fragmentation markedly impact the performance of computational phenotyping algorithms. Also, structured data might be more susceptible to misclassification biases depending on the clinical characteristics of the disease [51].

Our group developed automated chart review algorithms for asthma ascertainment with 97% sensitivity and 95% specificity [31]. This time saving automated chart review has improved the recognition and care of childhood asthma in the clinical setting and enables large-scale clinical studies [52].

A major strength of this study is the establishment of operational definitions for multiple AIMs enabling us to develop computational algorithms to identify AIMs which would enable us to study the association of asthma with AIMs on a large scale using EHRs.

A limitation of the study is the sample size. The number of expert panelists was relatively small compared with other Delphi studies, but data collection from clinician participants through the online questionnaire had a 100% response rate from clinical participants by sending frequent reminders. As the panel of experts were selected by convenience sampling, the sub-specialties of the core team, expert internal panel and external panel were not anonymous. However, the answers given on the online questionnaire were anonymous. In addition, the core team and internal expert panel members were all from one institution. To partially ameliorate this, we performed the external rounds in order to gain the generalizability for each definition as much as possible.

## Conclusion

The objective establishment of consistent, robust, and practical operational definitions of multiple AIMs through a modified Delphi technique is a key step towards developing reliable computational algorithms for automated chart review to mitigate the risks and poor outcomes of AIMs through asthma research and care.

## Supplementary Information

**Additional file 1**. A series of online questionnaires through which eight internal and 5 external expert panelists were invited to individually complete to provide judgement and feedback throughout three sequential internal rounds and two external rounds. This questionnaire was used sent to the external panelists for the final round.

## Data Availability

The datasets generated and/or analyzed during the current study are not publicly available as they include protected health information. Access to data could be discussed per the institutional policy after IRB at Mayo Clinic approves it. (Contact information: Juhn.young@mayo.edu).

## Abbreviations

AI: Artificial Intelligence
AIMs: Asthma-associated infectious and inflammatory multimorbidities
EHR: Electronic health record
EEP: External expert panel
ICD: International statistical classification of diseases
IEP: Internal expert panel
