# Supplementary material for: Establishing an expert consensus for the operational definitions of asthma-associated infectious and inflammatory multimorbidities for computational algorithms through a modified Delphi technique
Source: BMC Med Inform Decis Mak. 2021 Nov 8;21:310. doi: 10.1186/s12911-021-01663-y (PMC8573872; doi:10.1186/s12911-021-01663-y)
Supplement: Supplementary file 1 — Additional file 1. A series of online questionnaires through which eight internal and 5 external expert panelists were invited to individually complete to provide judgement and feedback throughout three sequential internal rounds and two external rounds. This questionnaire was used sent to the external panelists for the final round. [file 12911_2021_1663_MOESM1_ESM.pdf]

EXTERNAL Round TWO

Start of Block: INFECTIOUS DISEASES

JS

THE STUDY TEAM PROPOSES THE FOLLOWING OPERATIONAL DEFINITIONS FOR EACH CONDITION

INFECTIOUS DISEASES

Q1. **Invasive Bacterial Infection**

- Any pathogenic bacteria isolated from normally sterile body fluid including blood, CSF, pleural fluid, pericardia fluid, peritoneal fluid, or synovial fluid ***AND***
- Any physician diagnosis of sepsis, bacteremia, meningitis, encephalitis, mastoiditis, brain abscess, pneumonia, cellulitis, osteomyelitis, septic arthritis, pleuritis, or pericarditis, and pyelonephritis documented in medical records related to bacteria source cultured

Would you agree with this?

◯ Yes (1)

◯ No (2)

Display This Question:

If Q1 = 2

Q2 **Please suggest what specific changes you propose to Invasive Bacterial Infection:**

____

____

____

____

____

JS

Q3 **Frequent Streptococcus Pyogenes Upper Respiratory Infection**

- Physician diagnosis of sore throat, pharyngitis and tonsillitis ***AND***
- Throat swab test (rapid antigen detection test (RADT), Streptococcus pyogenes culture, or PCR [Polymerase chain reaction]) ***AND***
- 3 or more episodes within 12 months

Would you agree with this?

○ Yes (1)

○ No (2)

Display This Question:

If Q3 = 2

Q4 **Please suggest what specific changes you propose to Frequent Streptococcus Pyogenes Upper Respiratory Infection:**

____

____

____

____

____

JS

Q5. **Pneumonia**

- Physician diagnosis of pneumonia documented ***AND***
- Compatible exam findings (fever >=100.5◦F AND cough AND rale or crackle or crepitation) ***OR*** Chest radiograph finding suggesting pneumonia (consolidation, lobar pneumonia, and infiltrates)

Would you agree with this?

○ Yes (1)

○ No (2)

Display This Question:

If Q5 = 2

Q6 **Please suggest what specific changes you propose to Pneumonia:**

____

____

____

____

____

JS

Q7 **Recurrent or Persistent Otitis Media**

- CPT (Current Procedural Terminology) codes for tympanostomy tube placement (surrogate marker for either persistent or recurrent otitis media during childhood)

Would you agree with this?

○ Yes (1)

○ No (2)

Display This Question:

If Q7 = 2

Q8 **Please suggest what specific changes you propose to <u>Recurrent or Persistent Otitis Media</u>:**

____

____

____

____

____

JS

Q9 **<u>Recurrent or Persistent Infectious Sinusitis</u>**

- 4 or more episodes of Physician diagnosis of sinusitis documented with antibiotic prescription over 12 months ***AND***
- Sinus CT findings suggestive of sinus opacification or air/fluid level OR sinus surgery

Would you agree with this?

◯ Yes (1)

◯ No (2)

Display This Question:

If Q9 = 2

Q10 **Please suggest what specific changes you propose to <u>Recurrent or Persistent Infectious Sinusitis</u>:**

____

____

____

____

____

JS

Q11 **<u>Bordetella Pertussis</u>**

- PCR+ for Bordetella pertussis from the upper respiratory tract

Would you agree with this?

- ◯ Yes (1)
- ◯ No (2)

Display This Question:

If Q11 = 2

Q12 **Please suggest what specific changes you propose to <u>Bordetella Pertussis</u>:**

____

____

____

____

____

JS

Q13 **<u>Breakthrough Varicella Infection</u>**

- Physician diagnosis of Varicella (chickenpox) ***OR*** Positive lab result (PCR +) of Varicella infection occurred 42 days after varicella vaccination (excluding non-vaccinated children)

Would you agree with this?

- ◯ Yes (1)
- ◯ No (2)

Display This Question:

If Q13 = 2

Q14 **Please suggest what specific changes you propose to <u>Breakthrough Varicella Infection</u>:**

____

____

____

____

____

JS

Q15 **<u>Zoster (Shingles)</u>**

- Physician diagnosis of Herpes zoster ***AND***
- Positive lab result (PCR +) or anti-viral medication for Varicella zoster virus (eg, acyclovir)

Would you agree with this?

○ Yes (1)

○ No (2)

Display This Question:

If Q15 = 2

Q16 **Please suggest what specific changes you propose to <u>Zoster (Shingles)</u>:**

____

____

____

____

____

JS

Q17 **Urinary Tract Infection (UTI)**

- Urinary test results supporting the evidence of UTI Recovery of any organisms from a suprapubic specimen, at least 50 000 colony-forming units per milliliter (CFUs/mL) from a catheterized specimen, or at least 100 000 CFUs/mL from a clean-catch specimen ***AND***
- At least 10 white blood cells per microliter from an unspun specimen examined using a counting chamber or at least 5 white blood cells per high power field from a centrifuged specimen

Would you agree with this?

○ Yes (1)

○ No (2)

Display This Question:

If Q17 = 2

Q18 **Please suggest what specific changes you propose to Urinary Tract Infection (UTI):**

____

____

____

____

____

JS

Q19 **<u>Skin Fungal Infection</u>**

- Physician diagnosis of any skin fungal infection (except diaper rash) ***OR*** Fungal culture or fungal smear positive

Would you agree with this?

○ Yes (1)

○ No (2)

Display This Question:

If Q19 = 2

Q20 **Please suggest what specific changes you propose to <u>Skin Fungal Infection</u>:**

____

____

____

____

____

JS

Q21 **<u>Clinically Significant Viral Infection confirmed by lab</u>**

- A physician diagnosis of respiratory or gastrointestinal viral infection ***AND***
- PCR+ or culture+ test for respiratory or gastrointestinal virus infection

Would you agree with this?

○ Yes (1)

○ No (2)

Display This Question:

If Q21 = 2

Q22 **Please suggest what specific changes you propose to <u>Clinically Significant Viral Infection</u>:**

____

____

____

____

____

End of Block: INFECTIOUS DISEASES

Start of Block: INFLAMMATORY DISEASES

JS

THE STUDY TEAM PROPOSES THE FOLLOWING OPERATIONAL DEFINITIONS FOR EACH CONDITION

INFLAMMATORY DISEASES

Q23 **<u>Celiac Disease (CD)</u>**

- At least one diagnosis of Celiac disease documented by gastroenterologist **AND**
- (Positive CD serology markers (TTG IgA >10 higher than normal) AND (EMA positivity or DGA positively ***OR*** TTG IgA positivity AND Histologic findings (increased in IEL, villous atrophy, crypts hyperplasia)

Would you agree with this?

○ Yes (1)

○ No (2)

Display This Question:

If Q23 = 2

Q24 **Please suggest what specific changes you propose to <u>Celiac Disease (CD)</u>:**

____

____

Q25 **<u>Kawasaki Disease</u>**

- Physician diagnosis of Kawasaki disease documented at least once by infectious disease, cardiology, or rheumatology specialist

Would you agree with this?

○ Yes (1)

○ No (2)

Display This Question:

If Q25 = 2

Q26 **Please suggest what specific changes you propose to <u>Kawasaki Disease</u>:**

Q27 **<u>Appendicitis</u>**

- Surgeon's diagnosis in operation note (excluding incidental appendectomy or normal appendix) ***OR*** Physician diagnosis of appendicitis documented AND imaging study suggestive of appendicitis

Would you agree with this?

○ Yes (1)

○ No (2)

Display This Question:

If Q27 = 2

Q28 **Please suggest what specific changes you propose to <u>Appendicitis</u>:**

____

____

____

____

____

JS

Q29 **<u>Autoimmune Thyroiditis</u>**

- Physician diagnosis of autoimmune thyroiditis documented at least twice in a 6 month or greater span including endocrinologis's diagnosist at least once

Would you agree with this?

○ Yes (1)

○ No (2)

Display This Question:

If Q29 = 2

Q30 **Please suggest what specific changes you propose to <u>Autoimmune Thyroiditis</u>:**

____

____

____

____

____

Q31 **<u>Diabetes Type 1</u>**

- Physician diagnosis of Type 1 Diabetes documented at least twice in a 6 month or greater span including endocrinologist's diagnosis at least once

Would you agree with this?

○ Yes (1)

○ No (2)

Display This Question:

If Q31 = 2

Q32 **Please suggest what specific changes you propose to <u>Diabetes Type 1</u>:**

____

____

____

____

____

JS

Q33 **Diabetes Type 2**

- Physician diagnosis of Type 2 Diabetes documented at least twice in a 6 month or greater span including endocrinologist's diagnosis at least once

Would you agree with this?

○ Yes (1)

○ No (2)

Display This Question:

If Q33 = 2

Q34 **Please suggest what specific changes you propose to Diabetes Type 2:**

____

____

____

____

____

JS

Q35 **Inflammatory Bowel Disease (IBD; Crohn's Disease (CD), Ulcerative Colitis (UC))**

- Physician diagnosis of IBD, CD, or UC documented at least twice in a 6 month or greater span including gastroenterologist's diagnosis at least once

Would you agree with this?

○ Yes (1)

○ No (2)

Display This Question:

If Q35 = 2

Q36 **Please suggest what specific changes you propose to <u>Inflammatory Bowel Disease (IBD; Crohn's Disease (CD), Ulcerative Colitis (UC))</u>:**

____

____

____

____

____

JS

Q37 **<u>Juvenile Rheumatoid Arthritis (JRA), Juvenile Idopathic Arthritis (JA), or Rheumatoid Arthritis (RA)</u>**

- Physician diagnosis of JRA, JIA, and RA documented at least twice in a 6 month or greater span including a rheumatologist's diagnosis at least once

Would you agree with this?

○ Yes (1)

○ No (2)

Display This Question:

If Q37 = 2

Q38 **Please suggest what specific changes you propose to <u>Juvenile Rheumatoid Arthritis (JRA) or Rheumatoid Arthritis (RA)</u>:**

____

____

____

Q39 THANK YOU FOR COMPLETING THE SURVEY!

Please click SUBMIT to record your answers.

End of Block: INFLAMMATORY DISEASES
